# Placenta Previa, and Report of Cases

**Published:** 1896-06

**Authors:** John R. Shannon

**Affiliations:** Cabaniss, Ga.


### PLACENTA PREVIA, AND REPORT OF CASES.

By JOHN R. SHANNON, A.B., M.D.,

Cabaniss, Ga.

Placenta previa being an abnormal situation of the placenta, the question naturally arises, what is the cause of this abnormal condition? Placenta previa may be partial or complete; that is, the placenta may cover only a portion of the internal os uteri, or it may entirely cover it. The cause of this abnormal situation of the placenta has been a fruitful theme for discussion. We do not propose to enter into a detailed discussion of the causes. We know that the symptom which first warns us is the sudden flow of blood, which usually manifests itself during the last months of pregnancy. The hemorrhage, though not always, generally coincides with the menstrual period. The best authorities say that if the flow of blood does not endanger the life of the mother or child, the treatment should be directed to carrying the woman to full time. We have had two cases, one partial, one complete, and our experience leads us to the conclusion that the best thing to do after being satisfied that we have a placental presentation to deal with, is to terminate the pregnancy at once, more especially if it is a complete presentation. We should in no event wait longer than seven months, because at that period there is some chance for the child's life. Should we hesitate longer than this period, the danger both to the mother and child is constant and increasing, because a fatal hemorrhage is liable to occur at any time, and perhaps when medical aid cannot be secured, or if secured, too late to do any good.

In the spring of 1895 we were consulted by Mr. A. in regard to the condition of his wife. He reported that she was about five or six months advanced, and at a period about corresponding to the "monthly time" she had hemorrhage, which however was slight. About the seventh month the hemorrhage was somewhat more profuse. We were sent for, and after careful examination, satisfied ourselves that we had a case of complete placental presentation to deal with. Now the problem for us was, what is best for the mother and child? Should we attempt to carry her to full time or terminate the pregnancy? Because of the slight hemorrhage, we decided on the former plan, with this injunction, and its great importance impressed on the mind of the husband, should the flow become excessive, send for me at once. She was taken with labor pains in the afternoon and we were sent for early the next morning. We, in company with Dr. ——, called to see her. She complained of being very dizzy—could not see any distance. She had a severe hemorrhage during the night. We made instrumental delivery, but the patient never rallied, and died in one and a half hours after delivery. The cause of her death must have been the excessive loss of blood.

Case number two was one of partial presentation. This terminated successfully at full time without material trouble.

Should we ever have another case of complete placental presentation, we shall certainly advise immediate termination of pregnancy, and place the responsibility of refusal upon the patient.

---

### APPENDICITIS.

The question when to operate in appendicitis has always been a disputed one. Dr. Willy Meyer, of New York, says that in all cases of diffuse perforatvie appendicitis the operation must always be done at once. It is only exceptionally that patients recover without an operation. They have the best chance to recover if operated upon within the first twelve hours. In discussing acute appendicitis, he says the patients should always be carefully watched. If the pulse goes above 116 to 120, and has a tendency to stay there, this is an indication for an operation. In cases of doubt the operation is better than waiting.

In cases of subacute or mild attacks of appendicitis, also after the first severe attack, from which the patient recovers without immediate operation, the appendix should be removed. When the appendix has once been inflamed it must be regarded as a diseased organ, and one which is quite likely to give repeated, and more serious, even fatal, trouble in the future.—*Practical Medicine.*

